# Transcription factor encoding gene *OsC1* regulates leaf sheath color through anthocyanidin metabolism in *Oryza rufipogon* and *Oryza sativa*

**DOI:** 10.1186/s12870-024-04823-0

**Published:** 2024-02-28

**Authors:** Liqun Jiang, Shuwei Lyu, Hang Yu, Jing Zhang, Bingrui Sun, Qing Liu, Xingxue Mao, Pingli Chen, Dajian Pan, Wenfeng Chen, Zhilan Fan, Chen Li

**Affiliations:** 1 Rice Research Institute, Guangdong Academy of Agricultural Sciences, No. 3, Jinying East Road, Tianhe, Guangzhou, China; 2 Guangdong Key Laboratory of New Technology in Rice Breeding, No. 3, Jinying East Road, Tianhe, Guangzhou, China; 3 Guangdong Rice Engineering Laboratory, No. 3, Jinying East Road, Tianhe, Guangzhou, China; 4 Key Laboratory of Genetics and Breeding of High Quality Rice in Southern China (Co-construction by Ministry and Province), Ministry of Agriculture and Rural Affairs, No. 3, Jinying East Road, Tianhe, Guangzhou, China

**Keywords:** Anthocyanidin, Metabolic genome-wide association study, Leaf sheath color, OsC1, MYB transcription factor

## Abstract

Carbohydrates, proteins, lipids, minerals and vitamins are nutrient substances commonly seen in rice grains, but anthocyanidin, with benefit for plant growth and animal health, exists mainly in the common wild rice but hardly in the cultivated rice. To screen the rice germplasm with high intensity of anthocyanidins and identify the variations, we used metabolomics technique and detected significant different accumulation of anthocyanidins in common wild rice (*Oryza rufipogon*, with purple leaf sheath) and cultivated rice (*Oryza sativa*, with green leaf sheath). In this study, we identified and characterized a well-known MYB transcription factor, OsC1, through phenotypic (leaf sheath color) and metabolic (metabolite profiling) genome-wide association studies (pGWAS and mGWAS) in 160 common wild rice (*O. rufipogon*) and 151 cultivated (*O. sativa*) rice varieties. Transgenic experiments demonstrated that biosynthesis and accumulation of cyanidin-3-Galc, cyanidin 3-O-rutinoside and cyanidin O-syringic acid, as well as purple pigmentation in leaf sheath were regulated by *OsC1*. A total of 25 sequence variations of *OsC1* constructed 16 functional haplotypes (higher accumulation of the three anthocyanidin types within purple leaf sheath) and 9 non-functional haplotypes (less accumulation of anthocyanidins within green leaf sheath). Three haplotypes of *OsC1* were newly identified in our germplasm, which have potential values in functional genomics and molecular breeding of rice. Gene-to-metabolite analysis by mGWAS and pGWAS provides a useful and efficient tool for functional gene identification and omics-based crop genetic improvement.

**Supplementary Information:**

The online version contains supplementary material available at 10.1186/s12870-024-04823-0.

## Introduction

Rice, as one of the most important cereal crops [1] in Asia and Southeast Asia, including China, is higher demanded to be improved the quality under the premise of keeping the yield. In China, Guangdong province gets ahead in rice genetic breeding owe to the rich rice germplasm and the compatible climate for rice growth and development [2]. Abundant rice germplasm are the most important parent and gene resources for rice genetic breeding with three eternal themes of yield, resistance and quality [3]. Semi-dwarf breeding and hybrid rice breeding, which are known as the first and the second green revolution respectively, did both benefit from exploring and utilizing the excellent rice germplasm, such as the semi-dwarf rice variety ‘Aizaizhan’ and abortive common wild rice [4]. The demand of diversified cereals, like colored rice with high anthocyanidins accumulation, is growing for higher nutrients and people’s health, while common rice grain mainly contains many kinds of nutrient substance, such as water, carbohydrates, proteins, lipids, minerals and vitamins, within little anthocyanidins.

Anthocyanidins, a class of water-soluble flavonoids, are one of the largest groups of secondary metabolites in plants. Anthocyanidins can not only give distinctive floral organs (leaves, leaf sheath, hull, awn, and so on) various colors (purple, brown, or red), but also protect people from some chronic diseases, such as cancer, cardiovascular disease (CVD), non-alcoholic fatty liver disease (NAFLD), diabetes and obesity [5–9]. Besides, anthocyanidins play an important role in cleaning up reactive oxygen accumulated in plants upon various biotic and abiotic stress, such as ultraviolet (UV) radiation, infection by insects and pathogenic microorganism [10–14]. Based on the benefits of anthocyanidins, more and more biologists and breeders are committed to exploring the molecular mechanism of biosynthesis pathway and breeding new crop varieties which are rich in anthocyanidins.

Anthocyanidin biosynthesis is catalyzed by a class of enzymes, such as *CHS* (chalcone synthase), *CHI* (chalcone isomerase), *F3H* (flavanone 3-hydroxylase), *F3’H* (flavonoid 3’ hydroxylase), *DFR* (dihydroflavonol 4-reductase), *ANS* (anthocyanidin synthase) and *UFGT* (UDP-flavonoid glucosyl transferase), and regulated by a conserved MBW (MYB-bHLH-WD40) complex utilizing phenylalanine as a substrate [15, 16]. In *Arabidopsis thaliana*, MBW complex that activates the biosynthesis of anthocyanidins in vegetative tissues is demonstrated to be consist of MYBs of SG5 and SG6, basic helix-loop-helix subgroup, and WD40 repeat protein of TTG1 [16], whereas it comprising C1/Pl1 (R2R3-MYBs), R1/B1 (bHLHs), and PAC1 (WD40) in maize [17]. Although more and more traits which including grain size, panicle, callus induction, mesocotyl length, chlorophyll content, stigma exsertion, cold tolerance, drought tolerance had been examined by genome-wide association study [18] which was benefit from the fast development of genomic resequencing, the research on regulation of anthocyanidin biosynthesis used by GWAS in rice [19] behind and less than that in *A. thaliana* and maize. In rice, five putative regulators of anthocyanidin biosynthesis were identified and characterized by comparative mapping to the homologous nucleotide sequences of known orthologues in maize, including a R2R3-MYB gene *OsC1* and four bHLH genes, *Ra1*/*OsB1*, *Rb*, *Ra2* and *OsB2* [20–24]. The R2R3-MYB gene *OsC1* was demonstrated to be a determinant factor and a domestication-related gene of anthocyanidin biosynthesis in leaf sheath of cultivated rice [25, 26]. A ‘*C*-*S*-*A*’ gene system (*OsC1*-*OsB2*-*OsDFR*) was demonstrated to regulate hull pigmentation and reveal evolution of anthocyanidin biosynthesis pathway in rice [27]. In a word, although a few MYB and bHLH regulators have been identified and characterized in cultivated rice with the genetic variation analysis and evolution analysis of sequences between cultivated rice and wild rice, identification and characterization of these regulators in wild rice remain to be determined.

Although cultivated rice (*O. sativa*) hardly contains anthocyanidins and has green vegetative organisms, common wild rice (*O. rufipogon*), as the ancestor of cultivated rice, shows significant higher anthocyanidin accumulation and purple vegetative organisms (leaves, leaf blade, and leaf sheath). Screening the rice germplasm within higher accumulation of anthocyanidins and researching on anthocyanidins regulation, will be benefit for cultivating the cultivated rice varieties which are rich in anthocyanidins.

In order to screen the rice germplasm with high accumulation of anthocyanidins and identify the variations of related regulator(s) in rice germplasm in Guangdong province, in this study, phenotype of leaf sheath color and accumulation of anthocyanidins were respectively used to perform phenotypic and metabolic genome-wide association study (pGWAS and mGWAS). We have screened and identified 146 of 160 (91.25%) wild rice accessions and 12 of 151 (7.95%) cultivated rice varieties showed purple leaf sheath with significant higher accumulation of anthocyanidins, which could be the parent plants for hybrid rice breeding of anthocyanidin accumulation. Additionally, a well-known MYB transcription factor encoding gene, *OsC1*, was functionally characterized in our rice germplasm with three (two for the common wild rice and one for the cultivated rice) potential newly variations resulted in green leaf sheath and low accumulation of anthocyanidins. Exploring the regulation of anthocyanidin biosynthesis pathway in rice leaves would add insights into understanding the anthocyanidin biosynthesis pathway in rice grains.

## Materials and methods

### Plant materials and growth conditions

A collection of 311 rice accessions including 160 wild and 151 cultivated varieties (Supplementary Table S1) was used in this study. Plants were grown during the normal rice growing seasons in the field with normal agricultural practices in Hainan province, China [28]. Five leaves were collected from each of five randomly chosen plants at five-leaf stage as a sample, two biological replicate samples of each accession were used for metabolic and phenotypic genome-wide association studies.

### Metabolite profiling

A liquid chromatography-electrospray ionization-tandem mass spectrometry (LC-ESI-MS/MS) system was used for the relative quantification of widely targeted metabolites in freeze-dried rice leaf samples. The freeze-dried leaf samples were crushed using a mixer mill (MM 400, Retsch) with a zirconia bead for 1.5 min at 30 Hz, 100 mg dried powder was weighted and extracted overnight at 4 with 1.0 mL of 70% aqueous methanol containing 0.1 mg/L lidocaine (internal standard) for lipid-solubility metabolites or water-soluble metabolites [28–30]. Quantification of metabolites were carried out in a scheduled multiple reaction monitoring (MRM) mode. The relative signal intensities of the metabolites were standardized by firstly dividing them by the intensities of the internal standard and then log_2_ transforming them to generate the final data matrix.

### Genome-wide association analysis

Only SNPs with minor allele frequency (MAF) ≥ 0.05 and the number of varieties with a minor allele ≥ 6 in a (sub) population were used to carry out GWAS. Population structure was modeled as a random effect in Linear Mixed Model (LMM) using the kinship (K) matrix. We performed GWAS using LMM provided by FaST-LMM program [31]. The genome-wide significance thresholds (*P*_LMM_) was set to 2.61e-07 (0.05/191,487) after correction by the number of effective-independent SNPs [32], in which the 191,487 effective-independent SNPs for threshold calculation were obtained by using PLINK (10.1086/519795, 10.1038/nprot.2010.116) to remove the linkage disequilibrium SNPs.

### RNA extraction and sequencing

According to leaf sheath color and relative intensity of the three anthocyanidins, leaves of 10 wild rice accessions with highest anthocyanidins accumulation and purple leaf sheaths, and 10 cultivated rice accessions with lowest anthocyanidins accumulation and green leaf sheaths, were collected to extract total RNA and construct mRNA library for sequencing. Total RNA was isolated using trizol reagent (Invitrogen, Carlsbad, CA, USA) according to the manufacturer’s protocol. These cDNA libraries were amplified and sequenced on a BGISEQ-500 platform (BGI, Shenzhen, China). Raw reads including the adaptor sequences, low quality sequences, and unknown nucleotides were filtered into clean reads using standard quality control (QC) technique. The fragments per kilobase of transcript per million reads mapped (FPKM) method was used to calculate normalized expression levels using RNA-Seq by Expectation Maximization as previously described [33].

### Statistical analysis

The metabolite data of wild rice and cultivated rice accessions in this study comprise the means of three technical replications from the LC-MS/MS of one biological replicates. For each individual metabolite, the content was given as the average of the normalized metabolite levels in two replications. Metabolite data were log_2_ transformed to improve normality and normalized. The contents of three anthocyanidins in 311 rice accessions were used for hierarchical clustering analysis and visualization by R package heatmap version 1.0.12 (https://CRAN.R-project.org/package=pheatmap).

### Overexpression and knockout of *OsC1*

The over-expression construct of *OsC1* was generated by directionally inserting the full complementary DNA (cDNA) from wild rice accession DX386 into the vector pCAMBIA1300 under the control of the maize ubiquitin promoter. An sgRNA (5’-CTCCGGCCTAACATCAAGCG-3’) was designed and linked to pYLCRISPR/Cas9Pubi-H vector to generate *OsC1* knockout lines. Both the plasmids of overexpression and knockout of *OsC1* were introduced into *Agrobacterium tumefaciens* stain EHA105 to infect cultivated rice accession DX8 and wild rice accession DX386, respectively. A total of 22 and 16 transgenic positive plants (T0) were generated and named OE_*10350*_-1 to OE_*10350*_-22 and Δ_*10350*_-1 to Δ_*10350*_-16, respectively. After co-segregation tests, T1 progeny from three independent transgenic positive T0 plants for overexpression (OE_*10350*_-1 to OE_*10350*_-3) and knockout (Δ_*10350*_-1 to Δ_*10350*_-3) of *OsC1* were used for further analysis.

### Phenotype of transgenic lines

Three *OsC1* overexpression lines with the control plant DX8, and three *OsC1* knockout plants with the control plant DX386 were cultivated under the normal conditions with the same treatments for observing leaf sheath color and taking photos of the seedlings of all the transgenic lines and the controls.

### Quantitative real time polymerase chain reaction (qRT-PCR)

Total RNA was extracted from leaf sheath of *OsC1* overexpression plants and the control DX8 accession using RNA isolation kit (Magen). cDNA was generated in 25 µL reaction mixtures containing 2 µg DNase I-treated RNA, 200 U M-MLV reverse transcriptase (Takara), 40 U recombinant RNase inhibitor (Takara) and 0.1 µM oligo (dT)_18_ primer. RT-PCR was performed in total volumes of 10 µL containing 5 µL SYBR premix EX Taq (Takara), 0.2 µL Rox Reference Dye II (Takara), 0.4 mM gene-specific primers and 0.5 µL cDNA on an ABI 7500 real time PCR system (Applied Biosystems). The ubiquitin gene *Os03g234200* was used as an internal reference.

### DNA extraction and PCR identification

Genomic DNA was extracted from leaf sheath of *OsC1* mutation plants and the control DX386 accession using DNA extraction kit (TIANGEN). PCR was performed in total of 25 µL containing 12.5 µL Green Taq Mix (Vazyme), 1.0 µL DNA extraction, 1.0 µL *OsC1*-specific forward and reverse primers which are across the sgRNA. Fragments from PCR were cloned into pMD18-T vector and sequenced.

### Genome resequencing and haplotype analysis

Rice leaf samples of 160 wild rice and 151 cultivated rice accessions were collected to construct sequencing libraries according to the manufacturer’s instructions, and qualified libraries were sequenced using Illumina HiSeq platform. Quality of raw sequencing data were accessed using FastQC (v0.11.9) software [34]. Clean data were mapped onto reference genome (MSU7) using BWA (0.7.17-r1188) software with default parameter [35]. MarkDuplicates in Picard (2.12.1) was used to eliminate PCR duplication and sorting BAM files. All single nucleotide polymorphisms (SNPs), insertions and deletions (InDels) were called using HaplotypeCaller of Genome Analysis Toolkit (GATK, version 4.2.2.0) pipeline [36], and annotated using SnpEff (4.3 s) with the GFF3 file of MSU7 reference genome [37]. Software beagle (v5.2) was used to impute missing genetic variations that generated by GATK [38]. Although the accurate genomic phasing cannot be revealed by short reads sequencing, all genomic variations were still used for haplotyping *OsC1* by the jointing of SNPs and InDels with the consideration of heterozygous sites to help illustrating the whole genetic diversity of common wild rice. Genomic variations of selected genes were extracted based on the positions by using BCFTools [39]. Haplotype network of *OsC1* was constructed by our previously described method [40]. Haplotype network was constructed by Popart software [41].

## Results

### Analysis of leaf sheath color and anthocyanidin accumulation in *O. rufipogon* and *O. sativa*

**Fig. 1:**
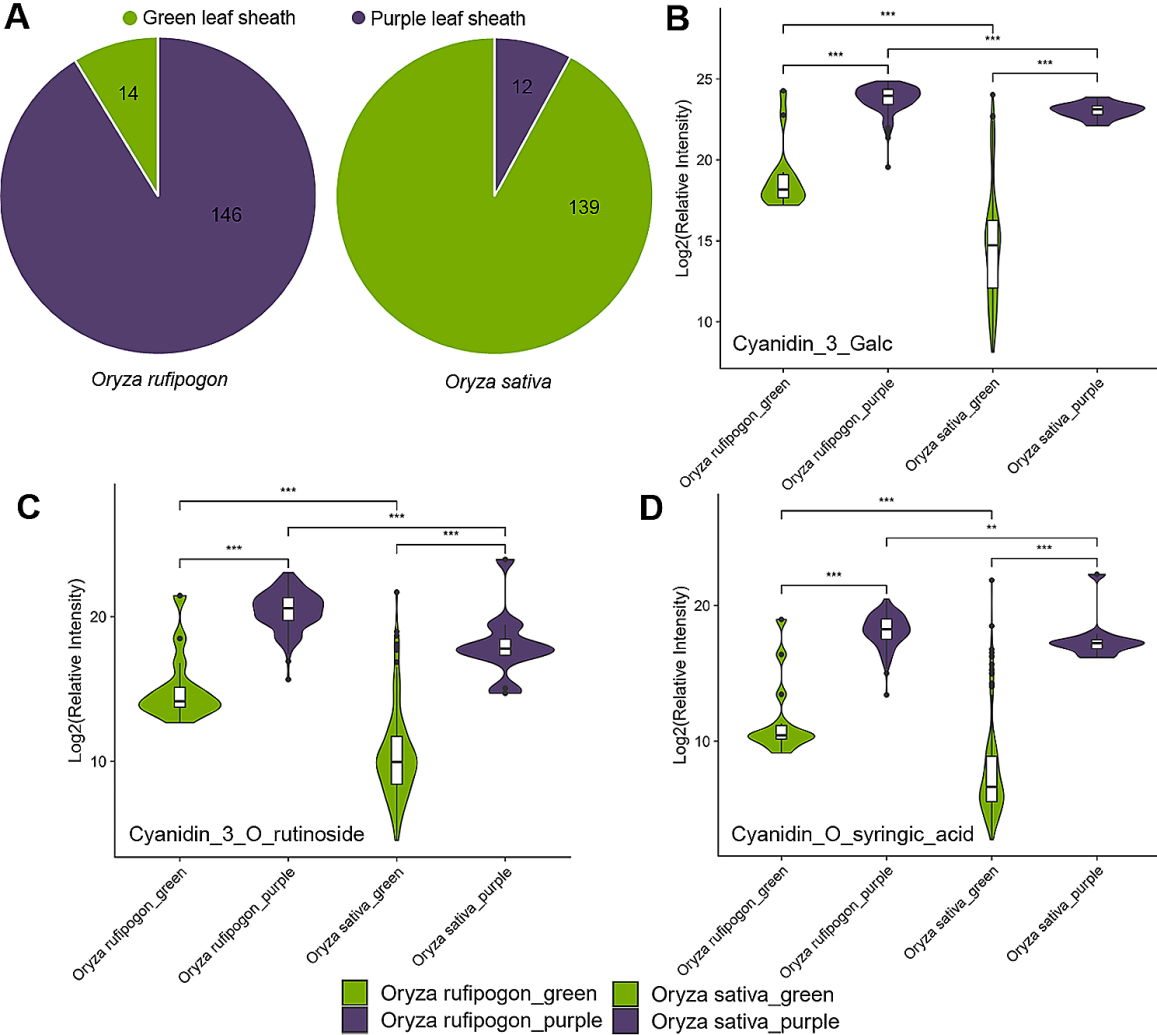
Phenotype of leaf sheath color and relative content of three anthocyanidins in the common wild rice (*Oryza rufipogon*) and the cultivars (*Oryza sativa*). **(A)** Count of accessions with purple and green leaf sheath. **(B)**, **(C)** and **(D)** Relative intensity of cyanidin-3-Galc, cyanidin 3-O-rutinoside, and cyanidin O-syringic acid. ‘***’ and ‘**’ indicate p-value<0.001 and 0.01, respectively

Significant difference in leaf sheath color between wild rice and cultivated rice was shown in Fig. 1A. 146 of 160 (91.25%) *O. rufipogon* accessions showed purple leaf sheath, while 139 of 151 (92.05%) *O. sativa* accessions showed green leaf sheath (Supplementary Table S1). To investigate whether the accumulation patterns of anthocyanidin or other metabolites were responsible for purple leaf sheath in *O. rufipogon* and *O. sativa*, a widely-targeted metabolomics method [28] based on liquid chromatography-electrospray ionization-mass spectrometry (LC-ESI-MS/MS) was applied into the comprehensive profiling analysis of anthocyanidin level in the leaves at five-leaf stage (termed ‘leaf’ hereafter) from the above rice accessions (Supplementary Table S1). Cyanidin-3-Galc, cyanidin 3-O-rutinoside and cyanidin O-syringic acid, established as colorant metabolites, were significantly higher accumulated in wild rice and showed 11.84-fold (*P* = 3.14E-52), 11.11-fold (*P* = 7.97E-20) and 4.60-fold (*P* = 2.47E-08) respectively, compared to cultivated rice (Fig. 1B and Table 1). Hierarchical clustering analyses (HCA) showed a visual normalized accumulation pattern, which showed the differences of relative content of these three metabolites in the two *Oryza* species (Supplementary Fig. S1). A series of correlation analyses showed positive correlation property (Pearson correlation, *R* = 0.82, 0.55, and 0.41, Student’s t-test *P*-value<0.0001, respectively) between each of the three anthocyanidin metabolites and purple leaf sheath (Table 1). Compared with *O. sativa*, which showed green leaf sheath within few accumulation of anthocyanidins, *O. rufipogon* showed purple leaf sheath with significant higher accumulation of anthocyanidins.

**Table 1:** Comparation of relative intensity of three anthocyanidins in *Oryza rufipogon* and *Oryza sativa*

Metabolite	Relative Intensity in Oryza rufipogon (Mean ± S.D.)	Relative Intensity in Oryza sativa (Mean ± S.D.)	Fold Change (Oryza rufipogon/ Oryza sativa)	R	*P*-value
Cyanidin-3-Galc	1.48E + 07 ± 7.73E + 06	1.25E + 06 ± 3.05E + 06	11.84	0.82	3.14E-52
Cyanidin 3-O-rutinoside	1.86E + 06 ± 1.69E + 06	1.67E + 05 ± 1.34E + 06	11.11	0.55	7.97E-20
Cyanidin O-syringic acid	3.54E + 05 ± 2.81E + 05	7.71E + 04 ± 5.22E + 05	4.60	0.41	2.47E-08

### mGWAS and pGWAS analysis on anthocyanidins accumulation in rice leaves

**Fig. 2:**
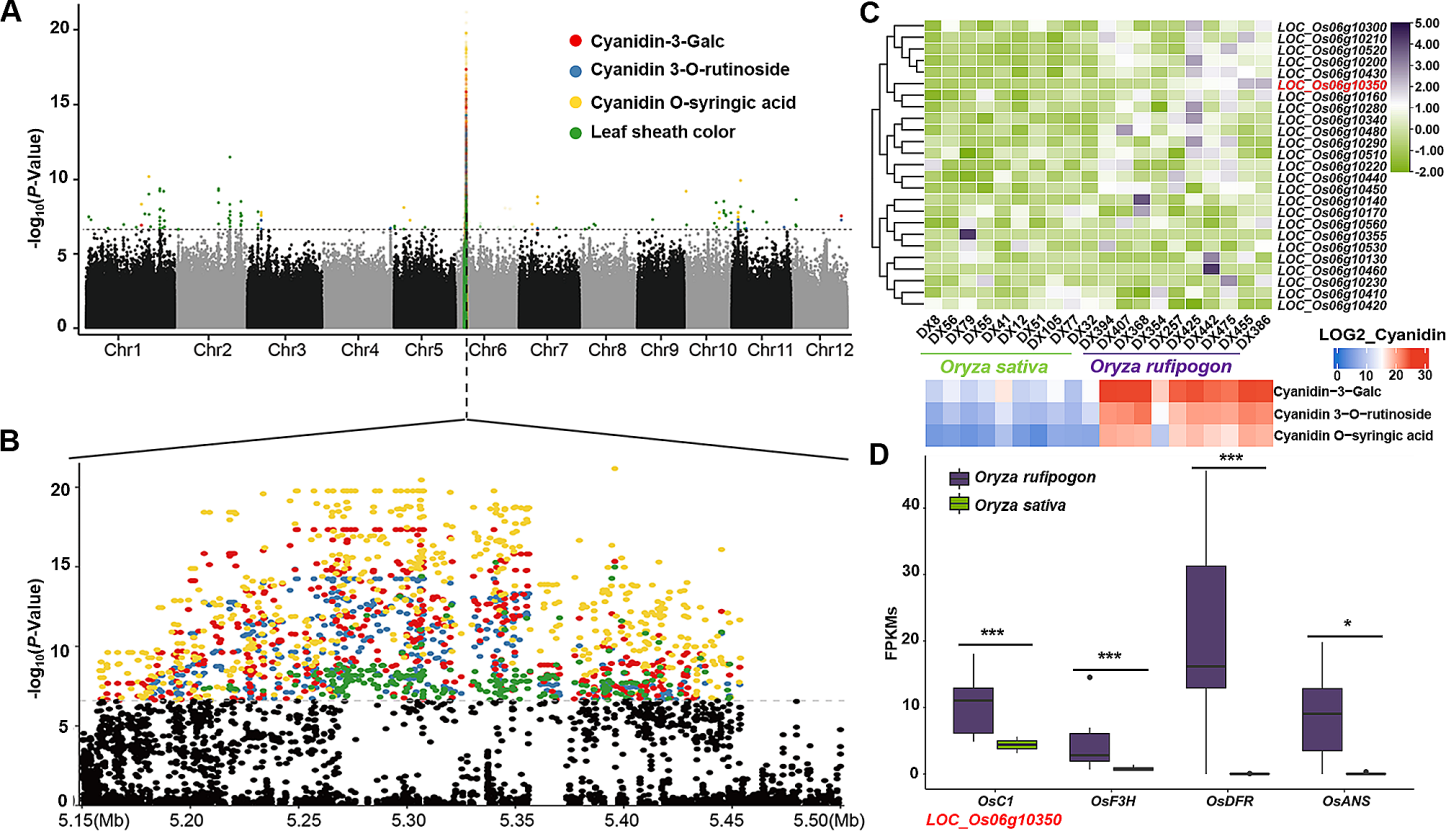
Mapping of *OsC1* using GWAS and expression analysis of anthocyanidins biosynthesis relative genes. **(A)** Manhattan plots for GWAS of 3 anthocyanidins traits and leaf sheath color across 12 rice chromosomes. The strength of association is indicated as the negative logarithm of the *P* value for the linear mixed model. All metabolite-/phenotype-SNP associations with *P* value below 2.61E-07 (horizontal dashed line) are plotted against the genome location in intervals of 1 Mb. **(B)** Regional Manhattan plot for 3 anthocyanidins traits and leaf sheath color trait in 5.15 Mb − 5.50 Mb region on chromosome 6. **(C)** Heatmap of 10 wild and cultivated rice accessions by normalized log2 of relative content of the three anthocyanidins and FPKMs of 25 candidate genes in the region located commonly by mGWAS and pGWAS. Candidate gene *OsC1* (*LOC_Os06g10350*) was noted by red font. **(D)** Expression analysis of *OsC1* and anthocyanidin biosynthesis relative genes (*OsF3H*, *OsDFR* and *OsANS*) in *Oryza rufipogon* and *Oryza sativa*. ‘***’ and ‘*’ indicate *p-value*<0.001 and 0.05 for t-test, respectively

mGWAS were performed for the three anthocyanidin metabolites in all the 311 rice accessions. The association results (Fig. 2A and B) showed that natural variations in cyanidin-3-Galc (lead SNP Chr6: 5,395,867 nt, *P*-value 4.63E-18), cyanidin 3-O-rutinoside (lead SNP Chr6: 5,272,133 nt, *P*-value = 4.77E-16) and cyanidin O-syringic acid (lead SNP Chr6: 5,395,867 nt, *P*-value = 6.77E-22) were significantly co-localized on Chr6: 4,212,610 nt − 5,665,639 nt. Not surprisingly, pGWAS on leaf sheath color showed a significant association on Chr6: 4,163,871 nt − 5,394,495 nt and within the lead SNP (5,394,495 nt, *P*-value = 1.71E-18) located closely to the lead SNPs of the three anthocyanidins.

To further screen the candidate gene, 10 wild rice accessions with purple leaf sheath and relative higher intensity of the three anthocyanidins, as well as 10 cultivated rice varieties with green leaf sheath and less accumulation of the three anthocyanidins, were used for RNAseq and transcriptome analysis. According to the rice genomic annotation, except for transposons and genes without expression in all selected samples, the remaining 25 genes (Supplementary Table S2) were located in a region which was shown in Fig. 2B. As shown in Figs. 2C and 11 of 25 candidate genes had higher FPKM values in *O. rufipogon* than that in *O. sativa*. Of 11 candidate genes, a well-known gene, *LOC_Os06g10350*, which is annotated as a MYB transcription factor and named *OsC1*, had been reported to be responsibility for accumulation of anthocyanidins and color of vegetative tissues in cultivated rice [26].

In addition, three anthocyanidin biosynthesis relative genes, *OsF3H*, *OsDFR*, and *OsANS*, showed the same expression tendency as *OsC1*, with significant (*P*-value = 0.01209, 0.000545, 0.000917 and 0.000751, respectively) higher FPKM values in wild rice accessions than that in cultivated rice varieties (Fig. 2D). This result showed the anthocyanidins biosynthesis in *O. rufipogon* may be regulated by the three downstream genes of *OsC1*.

### Functional characterization of *OsC1* in anthocyanidins biosynthesis in *O. rufipogon* and *O. sativa*

**Fig. 3:**
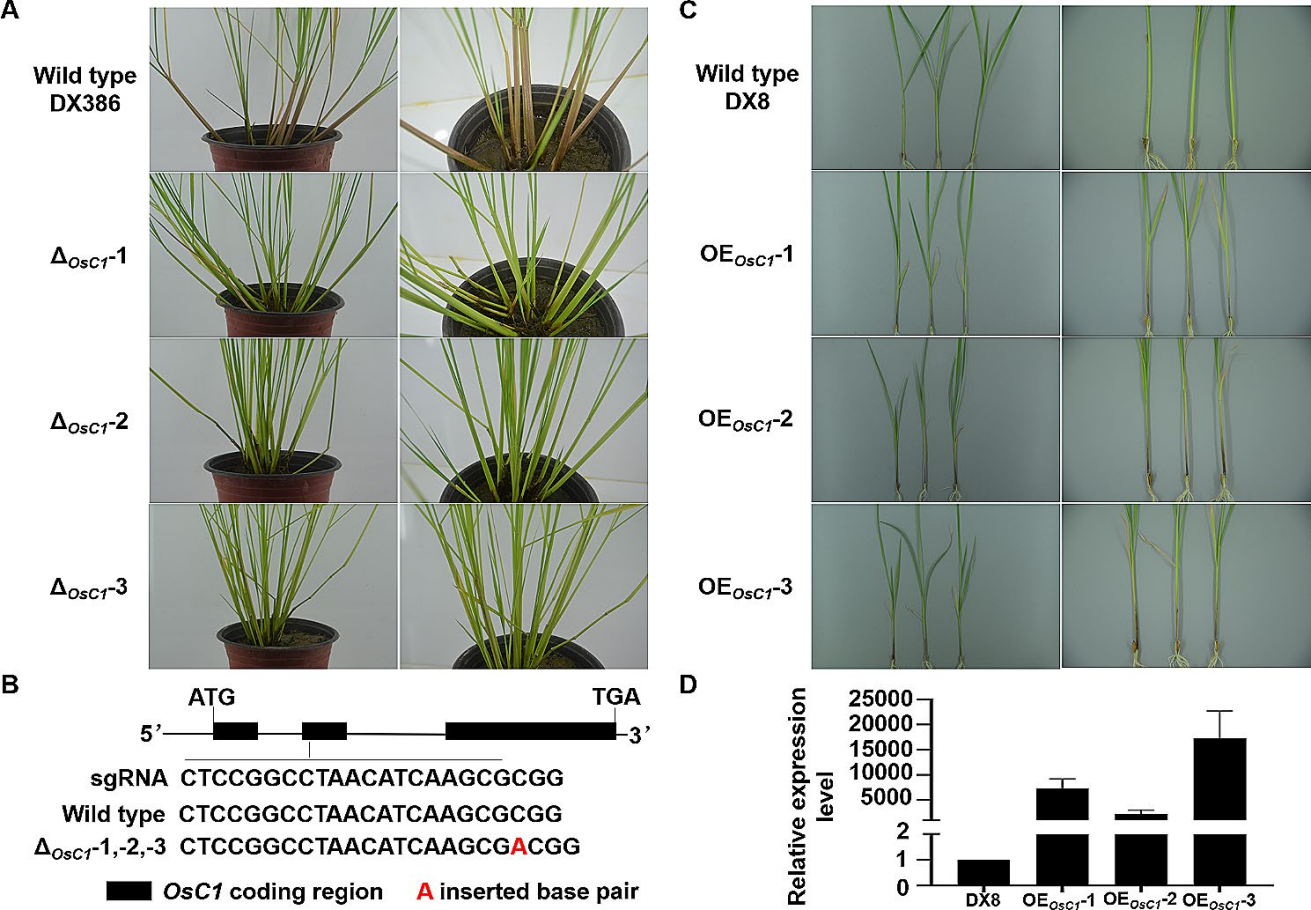
Functional analysis of *OsC1*. **(A)** Comparison of phenotype of leaf sheath color among wild type (*Oryza rufipogon* accession DX386) and three gene knockout lines (Δ_*OsC1*_-1, Δ_*OsC1*_-2, and Δ_*OsC1*_-3). **(B)** Sequences of *OsC1* in wild type and three gene knockout lines. **(C)** Comparison of phenotype of leaf sheath color among wild type (*Oryza sativa* accession DX8) and three gene overexpression lines (OE_*OsC1*_-1, OE_*OsC1*_-2, and OE_*OsC1*_-3). **(D)** Relative expression of *OsC1* in wild type and three gene overexpression lines by qRT-PCR

To investigate the native function of *OsC1* in *O. rufipogon* and *O. sativa*, we generated 3 mutants in DX386 (common wild rice) and DX8 (cultivated rice) backgrounds respectively. Three *OsC1* gene knockout (Δ_*OsC1*_-1, Δ_*OsC1*_-2, and Δ_*OsC1*_-3) and three overexpressed (OE_*OsC1*_-1, OE_*OsC1*_-2, and OE_*OsC1*_-3) lines were respectively verified by genome sequencing and qRT-PCR, respectively. As shown in Fig. 3A and B, compared to wild type *O. rufipogon* accession DX386 with purple leaf sheath and functional *OsC1* coding region, three *OsC1* gene knockout lines showed green leaf sheath and homozygous mutation of an ‘A’ base pair insertion at the position 69 of the second exon of *OsC1*. On the other hand, compared with the control *O. sativa* accession DX8 with green leaf sheath, overexpressed *OsC1* gene resulted in purple leaf sheath in the three overexpressed lines (Fig. 3C) with significant higher expression levels (Fig. 3D; respectively as 7284, *P*-value = 0.022; 2226, *P*-value = 0.034; and 17,251, *P*-value = 0.032, folds).

### Haplotype analysis of *OsC1* with anthocyanidins intensity and color variations in natural wild rice and cultivated rice germplasm

**Fig. 4:**
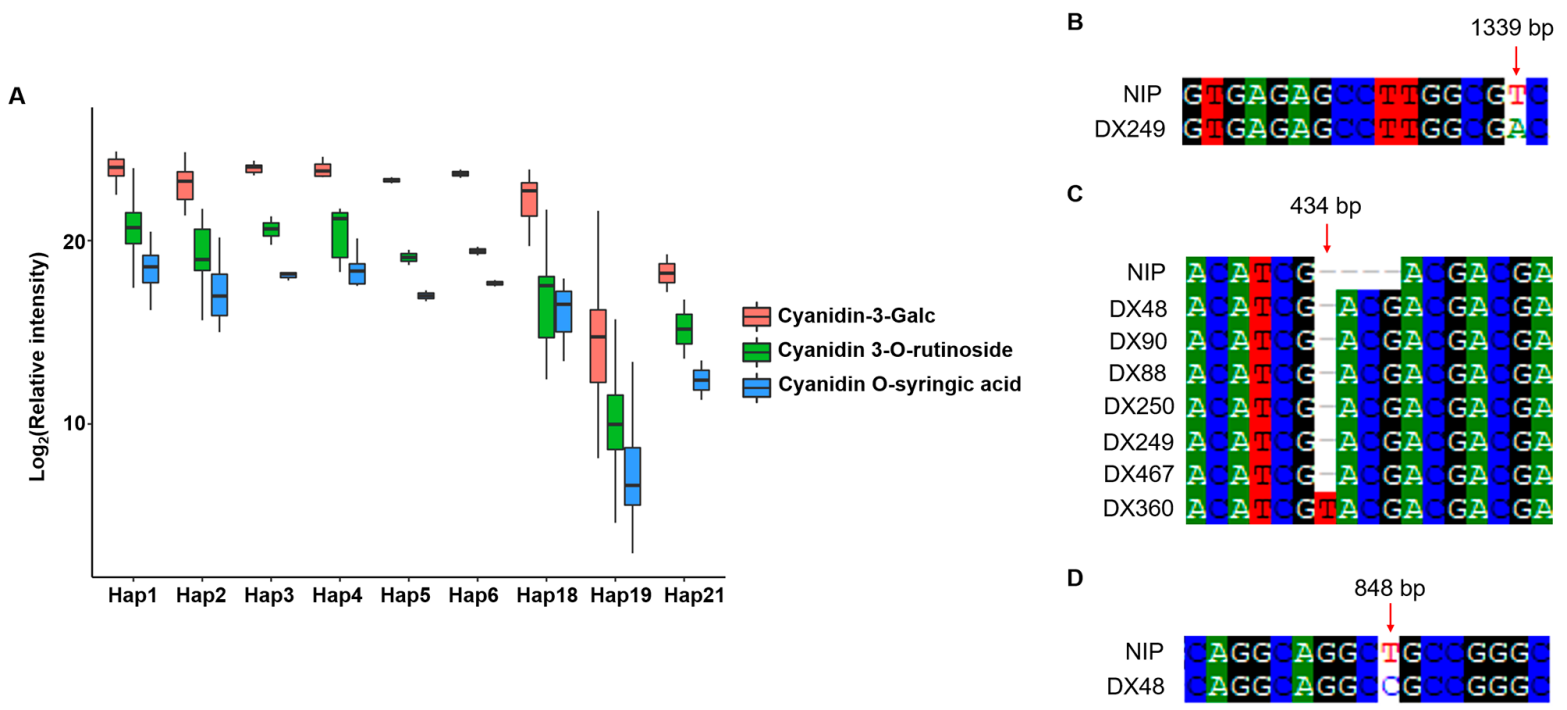
Relative content of the three anthocyanidins for 9 haplotypes in which contained at least two rice accessions and sequencing identification for the three potential new haplotypes of *OsC1*. **(A)** Relative intensity of the three anthocyanidins (cyanidin-3-Galc, cyanidin 3-O-rutinoside, and cyanidin O-syringic acid) of accessions with 7 functional haplotypes (Hap1-6, 18) and 2 non-functional haplotypes (Hap19, 21). **(B)**, **(C)** and **(D)** First generation sequencing identification of the three potential new haplotypes of OsC1 obtained through the whole genome sequencing. **(B)** Hap21, ‘T’ to ‘A’. **(C)** Hap22, ‘T’ insertion. **(D)** Hap24, ‘T’ to ‘C’

Since purple leaf sheath and relative higher accumulation of the three anthocyanidins were regulated by *OsC1*, we could test whether the color-producing and metabolite-accumulating model are universal among natural wild rice and cultivated rice germplasm by analyzing *OsC1* haplotypes. 25 genome sequence variations of *OsC1* were comprehensively analyzed in the total 311 rice accessions, combined with phenotypes of the leaf sheath color and the average relative intensity of the three anthocyanidins (Supplementary Table S3). 9 haplotypes (Hap1-6, 18, 19, 21) contained at least 2 rice accessions in each one and totally contained 295 of 311 rice accessions (Fig. 4A).

As shown in Supplementary Table S3 and Fig. 4A, in-depth analysis of *OsC1* revealed eighteen functional haplotypes (Hap1-18) with relative higher intensity of the three anthocyanidins and seven non-functional haplotypes (Hap19-25) with nearly no accumulation of the three anthocyanidins. Hap1 and Hap19 respectively represented the major functional and non-functional haplotypes which contained 107 (60.80% of anthocyanidin-abundant rice) and 128 (94.81% of anthocyanidin-absent rice) rice accessions, with only one difference (10 bp deletion, ‘-ACTGGAACAG-’) at the position from 881 nt to 890 nt of coding sequence of *OsC1*. All rice accessions in Hap19, including 6 wild rice and 122 cultivate rice varieties, consistently showed green leaf sheath without accumulation of the three anthocyanidins. 95.33% rice accessions in Hap1, including 101 wild rice and 1 cultivated rice varieties, also consistently showed purple leaf sheath with relative higher accumulation of the three anthocyanidins although 3 wild rice and 2 cultivated rice varieties showed unmatched phenotype (green leaf sheath with high accumulation of the three anthocyanidins). This result demonstrated that the variation (10 bp deletion) was the major determinant of color pigmentation and anthocyanidins accumulation. 18 of 21 rice accessions in Hap2 which had only one difference from Hap1 at the variation location 881 nt for a heterozygous genotype ‘T/T ACTGGAACAG’ also showed purple leaf sheath with relative higher intensity of the three anthocyanidins, except other 3 (1 wild rice and 2 cultivated rice varieties) showed green leaf sheath. Compared with Hap1, Hap3-17, which were consist of 27 wild rice accessions with different heterozygous genotype variations at different locations of DNA sequence of *OsC1*. In addition, we also found that 6 rice materials (Hap21-25) without 10 bp deletion showed green leaf sheath with few accumulation of the three anthocyanidins. Mutation from ‘T’ to ‘A’ (Hap21, missense variant, Fig. 4B), a ‘T’ insertion (Hap22, frameshift variant, Fig. 4C), and mutation from ‘T’ to ‘C’ (Hap24, missense variant, Fig. 4D) may be new haplotypes of *OsC1* for regulating purple pigmentation and anthocyanidins accumulation in rice leaf sheath.

## Discussion

Most modern cultivated rice (*O. sativa*) varieties present green vegetative tissues (leaf, leaf sheath and leaf margin) with few accumulation of anthocyanidins. On the contrary, most wild rice (*O. rufipogon*) plants, as the ancestor of cultivated rice, are rich in anthocyanidins and show various colors in different tissues [42]. In our research, 146 of 160 (91.25%) wild rice plants showed purple leaf sheath with significant higher accumulation of cyanidin-3-Galc, cyanidin 3-O-rutinoside and cyanidin O-syringic, than the cultivated rice accessions, most of which (125 of 151, 82.78%) showed green leaf sheath with significant less accumulation of the three anthocyanidins. Different colors of leaf sheath and significant different accumulation of the three anthocyanidins between *O. rufipogon* and *O. sativa* demonstrated that the characterization of purple leaf sheath was artificially threw away during breeding, along with reduced accumulation of anthocyanidins, that is a similar result and conclusion with previous studies [19, 25, 27].

Although development of the second generation of sequencing and application of genome-wide association study have rapidly promoted functional characterization of genes associated with complex traits in rice [43–45], the linkage imbalance of the genome and the imbalance of the population structure normally resulted in primary mapping [25] and false association between the objective phenotype and putative gene [46, 47]. Accurate identification of phenotype is an important factor that decide the efficiency of GWAS. In this study, a well-known gene regulating leaf sheath color, *OsC1*, was co-located accurately and confirmed mutually by combining mGWAS and pGWAS. Identification and quantitation of metabolites through widely targeted metabolites profiling, as a repeatable and verifiable indicator, enhanced the degree of accuracy of mGWAS. Additionally, application of multi-omics, such as mGWAS and pGWAS, could improve the efficiency of mapping genes through co-location of metabolites and phenotypes, and lay the foundation for analyzing genetic relationship between metabolites and phenotypes. Pigmentation, attributed to accumulation of anthocyanidins, occurred both in rice leaf sheath and grains. Although key genes and regulation pathway in rice grains are different from that in rice leaf sheath, the high-efficiency and accurate application in rice leaf sheath could be used for reference in rice grains. For example, based on the whole genome resequencing, identifying and classifying the phenotype of color in rice grain for pGWAS, detecting the anthocyanidins content for mGWAS, transgenic researching on the loci especially co-located by pGWAS and mGWAS.

In this study, we used mGWAS and pGWAS to fast and accurately identify *OsC1* as a regulator of three anthocyanidins biosynthesis based on natural population (160 wild rice accessions and 151 cultivated rice varieties), while Zheng et al. [19] used pGWAS of anthocyanin content based on a worldwide collection consisting of 533 cultivated rice accessions. *OsC1* had been initially identified by homology mapping in maize [26, 48–51]. Haplotype analysis showed that the major variation, ’10-bp’ deletion or presence at the position 881 nt – 890 nt, which was also found by Zheng et al. [19] and Sun et al. [27], could explain the difference of leaf sheath color and intensity of the anthocyanidins in 123 (81.46% of 151) cultivated rice varieties and 146 (91.25% of 160) wild rice accessions in this study. It has been reported that three kinds of indels were identified in which 10 bp deletion occurred in almost all *indica* varieties, whereas -TC and -GAG deletions mainly occurred in temperate *japonica* accessions in the non-functional haplotypes [27]. We found the majority variation as reported because our cultivated rice varieties were belonging to *indica* varieties in south China. Other two common variations were found by Zheng et al. (Hap4 and Hap5 in their article) and in our study (Hap 7 and Hap 18). In addition, we also found three types of variations (Fig. 4B), namely ‘T’ to ‘A’ mutation (Hap21), single ‘T’ insertion (Hap22), and ‘T’ to ‘C’ mutation (Hap24), as new haplotypes of *OsC1* for regulating purple pigmentation and anthocyanidins accumulation in rice leaf sheath. RiceNavi is a brilliant design for rice molecular breeding, which provide a highly efficient platform for the usage of genomic knowledge in rice breeding [52]. Artificial selection for the newly identified haplotypes of *OsC1* in breeding could be assisted by RiceNavi, which will facilitate the selection of rice varieties with lacked anthocyanidins. Anthocyanidins biosynthesis and accumulation in rice may be simultaneously regulated by other genes and pathways except *OsC1*, because there were still 2.5% wild rice and 9.27% cultivated rice accessions showed contradiction between the leaf sheath color and relative intensity of the anthocyanidins by the control of the major variation of *OsC1*.

## Conclusion

Metabolome analysis revealed that the significant higher accumulation of anthocyanidins was responsible for the change of leaf sheath color from green in *O. sativa* to purple in *O. rufipogon*, which is widely demonstrated to regulate the color of many plants. Combination of phenotypic and metabolic genome-wide association studies accurately and fast co-located a well-known MYB transcript factor encoding gene *OsC1* which was reported to responsible for coloration in various of rice tissues. Functional characterization of *OsC1* in our study not only revealed that *OsC1* regulates leaf sheath color both in *O. rufipogon* and *O. sativa*, but also verified a high accuracy and efficiency of multi-omics that applied to identify candidate genes related to traits. The present study provided more rice germplasm within high intensity of anthocyanidins and new potential variations of *OsC1* which could benefit for rice breeding and molecular mechanism in accumulation of anthocyanidins.

### Electronic supplementary material

Below is the link to the electronic supplementary material.

**Supplementary Table S1**. Natural accessions used in anthocyanidins profiling

**Supplementary Table S2**. FPKMs of 25 candidate genes in the common region located by mGWAS and pGWAS

**Supplementary Table S3**. Sequence polymorphism of different haplotypes of *OsC1*

**Supplementary Figure S1**. Hierarchical clustering analysis of relative differences of cyanidin-3-Galc, cyanidin 3-O-rutinoside and cyanidin O-syringic acid in *Oryza rufipogon* and *Oryza sativa*. The relative content of each bin was normalized to unit variance and visualized by color. Red indicates high anthocyanidins abundance; blue indicates low abundance

## Data Availability

The sequencing data was available at the NCBI repository with accession ID PRJNA934413.

## References

[1] Khush GS (2005). What it will take to feed 5.0 billion Rice consumers in 2030. Plant Mol Biol.

[2] Zhang J, Pan DJ, Fan ZL (2022). Genetic diversity of wild rice accessions (*Oryza rufipogon* Griff.) In Guangdong and Hainan Provinces, China, and construction of a wild rice core collection. Front Plant Sci.

[3] Atwell BJ, Wang H, Scafaro AP (2014). Could abiotic stress tolerance in wild relatives of rice be used to improve *Oryza sativa*?. Plant Sci.

[4] Wing RA, Michael DP, Zhang QF (2018). The rice genome revolution: from an ancient grain to Green Super Rice. Nat Rev Genet.

[5] Tsuda T (2012). Dietary anthocyanidin-rich plants: biochemical basis and recent progress in health benefits studies. Mol Nutr Food Res.

[6] Valenti L, Riso P, Mazzocchi A (2013). Dietary anthocyanidins as nutritional therapy for non-alcoholic fatty liver disease. Oxidative Med Cell Longev.

[7] Zhang Y, Butelli E, Martin C (2014). Engineering anthocyanidin biosynthesis in plants. Curr Opin Plant Biol.

[8] Vinayagam R, Xu B (2015). Antidiabetic properries of dietary flavonoids: a cellular mechanism review. Nutr Metabolism.

[9] Cerletti C, De Curtis A, Bracone F (2017). Dietary anthocyanidins and healthy: data from FLORA and ATHENA EU projects. Br J Clin Pharmacol.

[10] Lois R, Buchanan BB (1994). Severe sensitivity to ultraviolet radiation in an *Arabidopsis* mutant deficient in flavonoid accumulation. Planta.

[11] Jenkins GI, Christie JM, Fuglevand G (1995). Plant responses to UV and blue light: biochemical and genetic approaches. Plant Sci.

[12] Jiang C, Gao X, Liao L (2007). Phosphate starvation root architecture and anthocyanidin accumulation responses are modulated by the gibberellin-DELLA signaling pathway in *Arabidopsis*. Plant Physiol.

[13] Wang H, Fan W, Li H (2013). Functional characterization of dihydroflavonol-4-reductase in anthocyanidin biosynthesis of purple sweet potato underlies the direct evidence of anthocyanidins function against abiotic stresses. PLoS ONE.

[14] Nakabayashi R, Yonekura-Sakakibara K, Urano K (2014). Enhancement of oxidative and drought tolerance in *Arabidopsis* by overaccumulation of antioxidant flavonoids. Plant J.

[15] Winkel-Shirley B (2001). Flavonoid biosynthesis. A colorful model for genetics, biochemistry, cell biology, and biotechnology. Plant Physiol.

[16] Xu W, Dubos C, Lepiniec L (2015). Transcriptional control of flavonoid biosynthesis by MYB-bHLH-WDR complexes. Trends Plant Sci.

[17] Petroni K, Tonelli C (2011). Recent advances on the regulation of anthocyanidin synthesis in reproductive organs. Plant Sci.

[18] Wang Q, Tang JL, Han B (2019). Advances in genome-wide association studies of complex traits in rice. Theor Appl Genet.

[19] Zheng J, Wu H, Zhu HB (2019). Determining factors, regulation system, and domestication of anthocyanin biosynthesis in rice leaves. New Phytol.

[20] Hu J, Anderson B, Wessler SR (1996). Isolation and characterization of rice *R* genes: evidence for distinct evolutionary paths in rice and maize. Genetics.

[21] Hu J, Reddy VS, Wessler SR (2000). The rice *R* gene family: two distinct subfamilies containing several miniature inverted-repeat transposable elements. Plant Mol Biol.

[22] Reddy VS, Scheffler BE, Wienand U (1998). Cloning and characterization of the rice homologue of the maize *C1* anthocyanidin regulatory gene. Plant Mol Biol.

[23] Sakamoto W, Ohmori T, Kageyama K (2001). The *purple leaf* (*pl*) locus of rice: the *PlW* allele has a complex organization and includes two genes encoding basic helix-loop-helix proteins involved in anthocyanidin biosynthesis. Plant Cell Physiol.

[24] Saitoh K, Onishi K, Mikami I (2004). Allelic diversification at the *C* (*OsC1*) locus of wild and cultivated rice: nucleotide changes associated with phenotypes. Genetics.

[25] Huang X, Wei X, Sang T (2010). Genome-wide association studies of 14 agronomic traits in rice landraces. Nat Genet.

[26] Chin HS, Wu YP, Hour AL (2016). Genetic and evolutionary analysis of purple leaf sheath in rice. Rice.

[27] Sun XM, Zhang ZY, Chen C (2018). The *C*-*S*-*A* gene system regulates hull pigmentation and reveals evolution of anthocyanidin biosynthesis pathway in rice. J Exp Bot.

[28] Chen W, Gong L, Guo Z (2013). A novel integrated method for large-scale detection, identification, and quantification of widely targeted metabolites: application in the study of rice metabolomics. Mol Plant.

[29] Dresen S, Ferreiros N, Gnann H (2010). Detection and identification of 700 drugs by multi-target screening with a 3200 Q TRAP LC-MS/MS system and library searching. Anal Bioanal Chem.

[30] Matsuda F, Okazaki Y, Oikawa A (2012). Dissection of genotype-phenotype associations in rice grains using metabolome quantitative trait loci analysis. Plant J.

[31] Lippert C, Listgarten J, Liu Y (2011). FaST linear mixed models for genome-wide association studies. Nat Methods.

[32] Li MX, Yeung JMY, Cherny SS (2012). Evaluating the effective numbers of independent tests and significant p-value thresholds in commercial genotyping arrays and public imputation reference datasets. Hum Genet.

[33] Li B, Dewey CN (2011). RSEM: accurate transcript quantification from RNA-Seq data with or without a reference genome. BMC Bioinformatics.

[34] Andrews S. FastQC: a quality control tool for high throughput sequence data. 2010.

[35] Li H, Durbin R (2009). Fast and accurate short read alignment with Burrows-Wheeler transform. Bioinformatics.

[36] McKenna A, Hanna M, Banks E (2010). The genome analysis toolkit: a MapReduce framework for analyzing next-generation DNA sequencing data. Genome Res.

[37] Cingolani P, Platts A, Wang LL et al. A program for annotating and predicting the effects of single nucleotide polymorphisms, SnpEff: SNPs in the genome of *Drosophila melanogaster* strain *w*^1118^; *iso*-2; *iso*-3, 2012. 10.4161/fly.19695 PMC3679285 22728672

[38] Browning BL, Tian X, Zhou Y (2021). Fast two-stage phasing of large-scale sequence data. Am J Hum Genet.

[39] Li H (2011). A statistical framework for SNP calling, mutation discovery, association mapping and population genetical parameter estimation from sequencing data. Bioinformatics.

[40] Yu H, Li Q, Li Y (2021). Genomics analyses reveal unique classification, population structure and novel allele of neo-tetraploid rice. Rice.

[41] Leigh JW, Bryant D (2015). POPART: full-feature software for haplotype network construction. Methods Ecol Evol.

[42] Li D, Chen C (1993). The characteristics of two ecotypes of *O. Rufipogon* in China and ecological investigation. J South Agric.

[43] Chen W, Gao Y, Xie W (2014). Genome-wide association analyses provide genetic and biochemical insights into natural variation in rice metabolism. Nat Genet.

[44] Xie W, Wang G, Yuan M et al. Breeding signatures of rice improvement revealed by a genomic variation map from a large germplasm collection. Proceedings of the National Academy of Sciences, 2015, 112: 5411–5419. 10.1073/pnas.1515919112 PMC4593105 26358652

[45] Zhao H, Yao W, Ouyang Y (2015). RiceVarMap: a comprehensive database of rice genomicc variations. Nucleic Acids Res.

[46] Myles S, Peiffer J, Brown PJ (2009). Association mapping: critical considerations shift from genotyping to experimental design. Plant Cell.

[47] Lipka AE, Kandianis CB, Hudson ME (2015). From association to prediction: statistical methods for the dissection and selection of complex traits in plants. Curr Opin Plant Biol.

[48] Yue B, Cui KH, Yu SB (2006). Molecular marker-assisted dissection of quantitative trait loci for seven morphological traits in rice (*Oryza sativa* L). Euphytica.

[49] Fan FJ, Fan YY, Du JH (2008). Fine mapping of *C* (chromogen for anthocyanin) gene in rice. Rice Sci.

[50] Gao DY, He B, Zhou YH (2011). Genetic and molecular analysis of a purple sheath somaclonal mutant in *japonica* rice. Plant Cell Rep.

[51] Zhao S, Wang C, Ma J (2016). Map-based cloning and functional analysis of the chromogen gene *C* in rice (*Oryza sativa* L). J Plant Biology.

[52] Wei X, Qiu J, Yong KC (2021). A quantitative genomics map of rice provides genetic insights and guides breeding. Nat Genet.

