# Supplementary material for: Transcription factor encoding gene OsC1 regulates leaf sheath color through anthocyanidin metabolism in Oryza rufipogon and Oryza sativa
Source: BMC Plant Biol. 2024 Feb 28;24:147. doi: 10.1186/s12870-024-04823-0 (PMC10900563; doi:10.1186/s12870-024-04823-0)
Supplement: Supplementary file 4 — Supplementary Figure S1. Hierarchical clustering analysis of relative differences of cyanidin-3-Galc, cyanidin 3-O-rutinoside and cyanidin O-syringic acid in Oryza rufipogon and Oryza sativa. The relative content of each bin was normalized to unit variance and visualized by color. Red indicates high anthocyanidins abundance; blue indicates low abundance [file 12870_2024_4823_MOESM4_ESM.doc]

**Supplementary Material**

**Transcription factor encoding gene *OsC1* regulates leaf sheath color through anthocyanidin metabolism in *Oryza rufipogon* and *Oryza sativa*.**

**Jiang Liqun^†^, Lyu Shuwei^†^, Yu Hang^†^, Zhang Jing, Sun Bingrui, Liu Qing, Mao Xingxue, Chen Pingli, Pan Dajian, Chen Wenfeng, Fan Zhilan, Li Chen*.**

***Correspondence: Li Chen** [**(lichen@gdaas.cn)**](mailto:(lichen@gdaas.cn))

**1 Supplementary Figure**

**
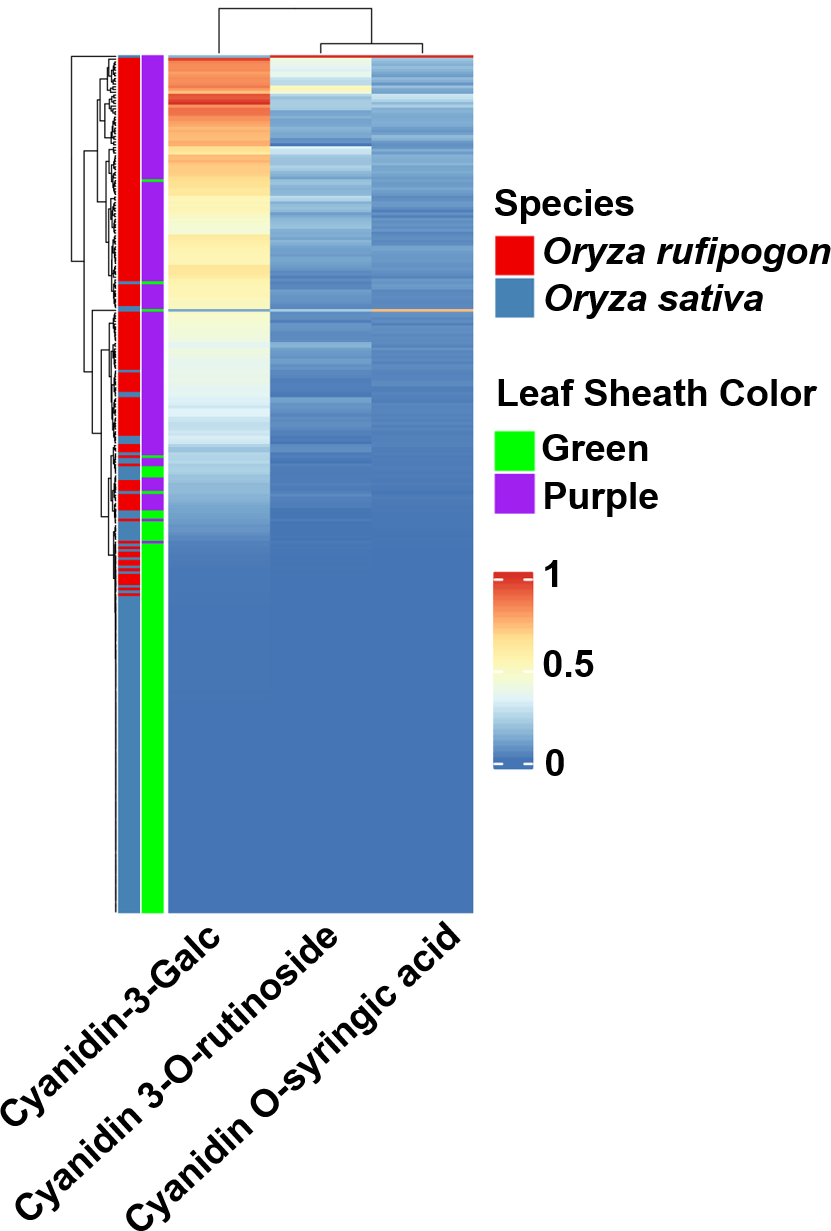
**

**Figure S1**. Hierarchical clustering analysis of relative differences of cyanidin-3-Galc, cyanidin 3-O-rutinoside and cyanidin O-syringic acid in *Oryza rufipogon* and *Oryza sativa*. The relative content of each bin was normalized to unit variance and visualized by color. Red indicates high anthocyanidins abundance; blue indicates low abundance.
